# The Apolipoprotein E Polymorphism rs7412 Associates with Body Fatness Independently of Plasma Lipids in Middle Aged Men

**DOI:** 10.1371/journal.pone.0108605

**Published:** 2014-09-30

**Authors:** M. Teresa Tejedor, Maria Pilar Garcia-Sobreviela, Marta Ledesma, Jose M. Arbones-Mainar

**Affiliations:** 1 Departamento de Anatomía, Embriología y Genética, Universidad de Zaragoza, Zaragoza, Spain; 2 Adipocyte and Fat Biology Laboratory (AdipoFat), Unidad de Investigación Traslacional, Instituto Aragonés de Ciencias de la Salud (IACS), Hospital Universitario Miguel Servet, Zaragoza, Spain; 3 Unidad de Prevención Cardiovascular, Instituto Aragonés de Ciencias de la Salud (IACS), Zaragoza, Spain; 4 CIBER Fisiopatología Obesidad y Nutrición (CIBERObn), Instituto Salud Carlos III, Madrid, Spain; University of Louisville, United States of America

## Abstract

**Background:**

The apolipoprotein E (*APOE*) gene is polymorphic, encoding one of 3 common alleles (ε2, ε3, ε4) produced from combinations of 2 non-synonymous SNPs (rs429358 and rs7412). APOE plays an important role controlling plasma lipids but its association with adipocyte functionality and body fatness remains to be determined.

**Methods:**

We analyzed fasting plasma lipids and genotyped the two main *APOE*-SNPs (rs429358 and rs7412), both located in the fourth exon of the *APOE*, in 4660 Caucasian middle-aged men free of cardiovascular disease.

**Results:**

The rs7412 SNP, which determines the APOE2 isoform, was significantly associated with Body Mass Index (BMI) and Waist Girth (WG) in a multivariate model accounting for age, smoking status and plasma lipids. BMI and WG were highest in TT homozygotes and lowest in CC homozygotes. This effect was independent of the rs429358 SNP, which failed to show any association with the BMI and WG variables. The odds ratio of being obese (BMI>30) for individuals carrying the APOε2 allele, present in 14% of the cohort and defined by the rs7412 SNP, was also significant in this multivariate model, with an OR of 1.27 (95% CI: 1.01–1.59).

**Conclusions:**

This study provides an evidence of a lipid-independent association between the APOE SNP rs7412 and body fatness surrogates, BMI and WG, in a large cohort of middle-aged males.

## Introduction

Obesity is a multifaceted disorder that increases the risk of developing cardiovascular disease and diabetes and is closely linked to disturbances in lipid and glucose metabolism [1]. Apolipoprotein E (APOE) binds to lipoprotein particles and mediates their catabolism, playing a crucial role in lipid regulation. The rs429358 and rs7412 single nucleotide polymorphisms (SNP) give rise to the ε2, ε3, and ε4 haplotypes of the human *APOE* gene.

Although mainly produced by the liver, APOE is also synthesized and secreted by adipose tissues [2]. Despite the pivotal role of APOE on lipid metabolism and adipocyte activity [3]–[5], genome-wide association studies (GWAS) have failed to detect an association of APOE genotype with human adiposity. (Reviewed in [6]). This is partially explained by i) the heterogeneity of the studied populations, ii) the low presence of subjects with the less common ε2 and ε4 haplotypes, and iii) the confounding impact that plasma lipids have on obesity: body fatness is positively related to circulating cholesterol and triglycerides [7] and, in turn, circulating cholesterol and triglycerides are modulated in a APOE isoform-dependent manner [8]. To overcome these caveats, we studied a large cohort of 4,660 Spanish middle-aged men free of Cardiovascular Disease to investigate whether *APOE*-SNPs, rs429358 and rs7412, are associated with body fatness measures, body mass index and waist girth, over and above the effect on plasma lipids.

## Methods

### Study participants

The Aragon Workers Health Study (AWHS) is a longitudinal study of the workers of the General Motors Spain automobile factory in Figueruelas (Zaragoza, Spain) [9]. Workers were excluded from the cohort if they have clinically overt CVD. The study was approved by the local Institutional Review Board (Comité Ético de Investigación Clínica de Aragón, CEICA) and participants provided written informed consent.

### Anthropometry and plasma biochemistry

Height and weight were determined using a standard steel strip stadiometer (SECA-440. SECA, Hamburg, Germany) and a digital electronic scale (SECA-778. SECA), respectively. Waist circumference was measured between iliac crest and the lower rib margin with flexible anthropometric tape (GulicK II -67019. Country Technologies, Gays Mills, WI. USA). Fasting serum triglycerides, total cholesterol and HDL-cholesterol were assayed by spectrophotometry (Chemical Analyzer ILAB 650, Instrumentation Laboratory).

### Genotyping

DNA from buffy coat was isolated using the FlexiGene DNA AGF3000 kit (Qiagen, Valencia, CA, USA) on an AutoGenFlex 3000 workstation (Autogen, Holliston, MA. USA) and genotyping was carried out in the Genetics Unit-Parque Cientifico de Madrid (Madrid. Spain). Briefly, samples were spotted onto 384 plates using a Beckman BioMek 2000 automated liquid handler (Beckman High Wycombe, UK) and diluted in a mix consisting in TaqMan Genotyping MasterMix (Applied Biosystems, Foster City, California)) and a mixture of pre-made TaqMan SNP genotyping assays; C_3084793_20 (rs429358) and C_904973_10 (rs7412) (Applied Biosystems). qPCR reactions were made in a HT7900 Fast Real-Time PCR System (Applied Biosystems). SDS 2.4 software (Applied Biosystems) was used for genotype calling.

### Statistical analysis

SNP & Variation Suite 7 Genetic Analysis Software v7.7.8 (Golden Helix, Bozeman, MT, USA) estimated allele frequencies, assessed Hardy-Weinberg equilibrium (HWE) and calculated linkage disequilibrium. Haplotype frequencies and individual haplogenotypes were estimated from genotypes by means of the expectation-maximization (EM) algorithm implemented in this software. We used the General Linear Model (GLM) in IBM SPSS v.19 (SPSS, Chicago, IL. USA) to fit different models comparing BMI and WG across *APOE* polymorphisms.

## Results

The polymorphisms rs429358 and rs7412 were genotyped in 4660 individuals of the AWHS cohort. Allele frequencies for rs429358 were 0.903 and 0.097 for T and C alleles, respectively. For rs7412, allele frequencies for C and T alleles were 0.939 and 0.061, respectively. Both SNPs showed no significant departure from Hardy-Weinberg Equilibrium (*P* = 0.880 and *P* = 0.371 for rs429358 and rs7412, respectively). Linkage disequilibrium between both SNPs was very low in these samples (r^2^ = 0.006). Combinations of SNP rs7412 and rs429358 determine *APOE* isoforms and, although four haplotypes were possible, only three were found: ε2 (6.1%), ε3 (84.2%) and ε4 (9.7%). We also compared the single-SNP results with the standard ε2, ε3, and ε4 diplotype models. The distribution of these genotypes in the cohort population was ε3/ε3 (71.0%), ε3/ε4 (16.3%), ε2/ε3 (10.1%), ε2/ε4 (1.2%), ε4/ε4 (0.9%), and ε2/ε2 (0.5%).

Detailed description of the AWHS cohort has been previously provided [9]. Information regarding plasma lipid biochemistry and smoking status of the participants is detailed in Table 1. In a crude regression model, higher BMI and WG were inversely associated with smoking and plasma HDL and directly correlated with circulating cholesterol, TG, being an ex-smoker and age (p<10^−5^ for all variables, in both fatness measures). A significant association was also observed between both the APOE polymorphism rs7412 as well as the rs429358 with circulating plasma cholesterol and triglycerides (Table 2).

**Table 1:** The Aragon Workers Health Study (AWHS) baseline characteristics.

Variable	N	Mean (S.D.)
Age (years)	4881	49.2 (8.7)
Weight (kg)	4854	81.6 (11.6)
Height (cm)	4870	171.5 (7.2)
Waist Girth (cm)	4816	97.2 (10)
Triglycerides (mg/dl)	4881	148.5 (107.1)
Cholesterol (Total) (mg/dl)	4881	212.9 (38.1)
Cholesterol (HDL) (mg/dl)	4881	52.5 (11.1)
BMI (kg/cm^2^)	4854	27.7 (3.6)
Smoking status	4842	
Non smoker	1653	34.2%
Ex-smoker	1420	29.3%
Smoker	1769	36.5%

**Table 2:** Apolipoprotein E genotypes and means (SD) of circulating lipids in the Aragon Workers Health Study.

	N (%)	Triglycerides	Cholesterol (Total)	HDL
		(mg/dl)	mg/dl)	(mg/dl)
**rs429358**				
N		4660	4660	4660
T/T	3799 (81.5)	147 (106.9)	211.8 (37.7)	52.6 (11)
C/T	818 (17.6)	152.9 (102.2)	216.9 (38.5)	51.6 (11)
C/C	43 (0.9)	190.3 (153)	223.8 (38.6)	49.6 (12.6)
P value*		0.009	<0.001	0.353
**rs7412**				
N		4660	4660	4660
T/T	4110 (88.2)	165.9 (82.9)	178.8 (29.5)	54.8 (15.9)
C/T	529 (11.4)	164.3 (97.2)	204.3 (40.3)	52.6 (11)
C/C	21 (0.5)	146.3 (107.8)	214.41 (37.4)	52.4 (11)
P value*		0.006	<0.0001	0.288

*P-values for the difference within each SNP including age as covariate.

We next explored the contribution of each SNP to body fatness beyond their effects on plasma lipids. Multiple regression analyses considering SNP rs7412 or rs429358 as independent variables and either BMI or WG as dependent variables were used. After adjustment for variables that affect body weight (i.e. smoking status, plasma lipids, and age) the rs7412 genotype was significantly associated with BMI (*P* = 0.018) as well as WG (*P* = 0.048). Thus, individuals carrying the T/T genotype displayed both higher BMI and larger WG followed by C/T and C/C (Table 3). On the contrary, the same regression model did not reveal significant associations with either BMI or WG and the rs429358 SNP. We additionally tested for an interaction (epistasis) between rs7412 and rs429358 in our regression models and found P-values for the interaction term of 0.331 for BMI and 0.229 for WG. This precluded a possible epistasis between those SNPs and suggested that rs7412 acts independently on body fatness.

**Table 3:** Association between individual APOE SNPs, rs429358 and rs7412, with body fatness.

	BMI (kg/m^2^)	Waist Girth (cm)
rs429358		
N	4597	4569
T/T	28.47 (0.29)	98.67 (0.79)
C/T	27.89 (0.23)	97.81 (0.63)
C/C	27.29 (0.53)	96.86 (1.47)
P value	0.789	0.980
rs7412		
N	4597	4569
T/T	29.55 (0.851)	100.59 (2.32)
C/T	28.17 (0.237)	98.46 (0.64)
C/C	27.53 (0.183)	96.99(0.50)
P value	0.018	0.048

Means and standard error of the means (SEM) of Body Mass Index (BMI) and waist girth values, adjusted for age, smoking status and lipid values.

**Figure 1:**
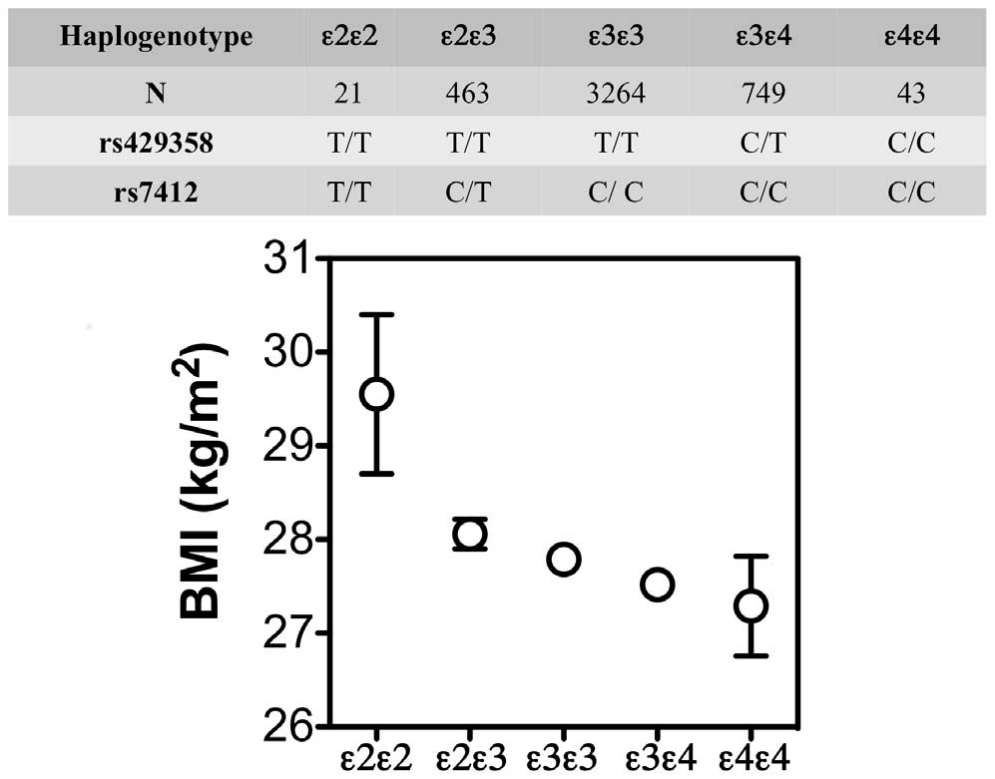
Mean adjusted BMI and standard error for five haplogenotype categories.

Multiple regression analyses involving classic APOE genotypes as independent variables and either BMI or WG as dependent variables revealed a significant effect of haplogenotypes on BMI after adjustment for age, lipids and smoking (*P* = 0.017) (Figure 1). *A posteriori* contrasts found a significant difference between ε2ε2 and non-ε2 carriers (ε3ε3, ε3ε4 and ε4ε4, *P* = 0.038, *P* = 0.018 and *P* = 0.024, respectively), with the ε2ε2 genotype associated with higher BMI. It should be noted that the ε2ε2 genotype includes the T/T genotype for rs7412, which was associated to higher BMI values, as previously stated. Using this model, the odds ratio of being obese (BMI>30) for individuals bearing a ε2 allele (13.4% of the cohort) was 1.27 (1.01–1.59, p = 0.035), when compared with those bearing no copies of this allele.

## Discussion

The human *APOE* locus (19q13.2) encodes a polymorphic protein of 299 amino acids with three haplotypes; ε2, ε3, and ε4, produced from combinations of two rs429358 and rs7412 coding non- synonymous SNPs. These haplotypes give rise to three homozygous (ε2ε2, ε3ε3, ε4ε4) and three heterozygous genotypes (ε2ε3, ε2ε4, ε3ε4). Several studies have supported linkage to obesity phenotypes at chromosome 19q13, where APOE gene is located. Thus, as early as 2002, a genome scan based on German families with obese children described an association between a region very close to 19q13 and obesity in children and adolescents [10]. Subsequent imprinted linkage analyses confirmed this suggestive connection between BMI and the 19q13 locus [11], [12].

In agreement with our results, APOE ε2 carriers (ie, carriers of the minor allele of rs7412) were described to have higher plasma APOE concentration as compared with subjects with ε3 and ε4 genotype, being plasma APOE concentration directly correlated with BMI [13]. Likewise, the ARIC Study reported that the APOE isoforms are associated with increasing BMI in the order: ε2>ε3>ε4 [14]. However, this relation is controversial with studies showing associations of the ε2 allele with increased BMI [14]–[17] or the ε4 allele with decreased BMI measures [18], [19], while others showed the ε4 genotype to be associated with obesity [20], [21]. A recent study showed no association between APOE polymorphisms and body fat mass in obese children [22], but another study described lower BMI in 8-year-old children carrying APOE4 [23]. We hypothesize that these seemly conflicting results could be explained by the confounding effects of plasma lipids when the relationship between APOE and adiposity is investigated. In this vein, a recent study failed to show any significant effect of the interaction between APOE genotype and BMI on blood lipid levels [24]. However, the predictive power of the regression model for LDL-C improved when gene-BMI interaction and gene-BMI interaction plus gene-nutrient interaction were added [24]. The authors reasoned that sample size of the study was relatively small (∼1000 individuals), perhaps affecting its ability and power to detect the effect of such interactions on lipid levels.

Associations between APOE isoforms and circulating lipid concentrations were described more than 30 years ago [25], and is also well known that these lipids are, in turn, associated with obesity [7]. Once lipid effects were normalized across genotypes we showed that the *APOE*-SNP rs7412 had a lipid-independent effect on body fatness. One may argue that controlling for lipids, which might be on a causal pathway between exposure (*APOE*-SNPs) and outcome (body fatness) could lead to an over-adjusted model. However, this would be the case if the only causal link between APOE and fatness was *via* plasma lipid modulation. In this regard, a number of biological pathways have been described in previous reports by us and others that suggest a lipid-independent role of APOE in the modulation of adipocyte function. These APOE isoform-dependent effects have been demonstrated *in vivo* as well as *in vitro* models [4], [5], [20], [26]. It can also be argued that BMI might not completely capture obesity status and WG is a more adequate measure of abdominal fat and total fat. In this sense, the fact that APOE-effect acts not only upon BMI but WG also lends support to the existence of APOE-dependent lipid-independent mechanisms regulating adipose tissue mass and thus obesity.

Our results also support the notion that, while circulating cholesterol and TGs are determined by both APOE SNPs acting synergistically, the rs7412 SNP affects BMI and WG independently of rs429358. This is of particular interest in regards to the 12.4% of the AWHS cohort individuals in whom a single SNP determination (i.e. the presence of a single T allele in the SNP rs7412) can inform about their obesity risk.

Our study has some inherent limitations. Due to its cross-sectional design, we cannot draw conclusions about causal relations between *APOE*-SNPs and fatness measurements. We do know, however, that the relation is independent of APOE effects on plasma lipids and relies on the rs7412 SNP. Additionally, since we focused on middle-aged males we cannot generalize our results to all adult populations. The strength of this study is that the large and homogeneous sample size of the AWHS Study allows a valuable opportunity for in-depth analysis of *APOE* allele effects in Caucasian middle-aged men free of CVD. This demographic is specially interesting as it represents a growing population of individuals who have adopted the food and lifestyle patterns of developed countries and are at high risk of developing metabolic disease. Our findings warrant further investigation in women, as well as in more heterogeneous populations, and underscore the importance of APOE in human metabolism beyond its role on lipoprotein regulation.

## Conclusions

Polymorphisms rs429358 and rs7412 in the human *APOE* gene are strongly associated with the variation of cholesterol and triglyceride levels in plasma. Further, once lipid variation is normalized across genotypes, the SNP rs7412 is also associated with body weight pointing towards the existence of a lipid-independent effect of APOE on body fatness.
